# Small molecule inhibitors target multiple neuropathological signaling to exert novel neuroprotection in intracranial aneurysms

**DOI:** 10.3389/fphar.2024.1469211

**Published:** 2024-11-07

**Authors:** Acharya Balkrishna, Shalini Mishra, Maneesha Rana, Satyendra Kumar Rajput, Suhrud Pathak, Keyi Liu, Muralikrishnan Dhanasekaran, Vedpriya Arya, Shalini Singh

**Affiliations:** ^1^ Patanjali Herbal Research Department, Patanjali Research Foundation, Haridwar, India; ^2^ Department of Pharmaceutical Sciences, Gurukula Kangri (Deemed to be University), Haridwar, India; ^3^ Department of Drug Discovery and Development, Harrison College of Pharmacy, Auburn University, Auburn, AL, United States

**Keywords:** intracranial aneurysm (IA), small molecule inhibitors (SMIs), pathophysiology, extracellular matrix, inflammation

## Abstract

Intracranial aneurysms (IAs) represent a critical health concern due to their potential to rupture, leading to severe morbidity and mortality. Small molecule inhibitors (SMIs) have emerged as promising therapeutic candidates for managing IA progression and rupture risk. The current landscape of SMIs targets various molecular pathways implicated in IA pathogenesis, including inflammation, endothelial dysfunction, and extracellular matrix (ECM) degradation. Among the prominent therapeutic candidates discussed are statins, recognized for their multifaceted effects, anti-inflammatory properties, and enhancement of endothelial stability, which may mitigate IA progression. Matrix metalloproteinase inhibitors are also highlighted for their role in preserving ECM structural integrity, essential for preventing IA wall weakening and rupture. Furthermore, the review evaluates the efficacy of anti-inflammatory agents such as corticosteroids and cytokine inhibitors in attenuating IA growth driven by inflammatory processes. Our findings highlight the possibility of several pharmaceutical therapies that target matrix remodeling, inflammation, and other underlying processes to manage cerebral aneurysms. By precisely delivering therapeutic chemicals, such as antioxidants, gene therapy vectors, or anti-inflammatory medicines, to the aneurysm site, these SMI technologies treat the underlying pathophysiological causes while sparing healthy brain tissue. This review underscores the potential of SMIs as adjunctive or primary therapies in the comprehensive management of IAs, emphasizing the need for further clinical research to optimize their efficacy and safety in clinical practice.

## 1 Introduction

Intracranial aneurysms (IAs) are pathological dilations of cerebral arteries characterized by a weakened vessel wall, posing a significant risk of rupture and subsequent subarachnoid hemorrhage (Wang et al., 2023). IAs are relatively prevalent, with a frequency of around 4% (Keedy, 2006). While typically asymptomatic, the rupture of an IA leading to Subarachnoid hemorrhage (SAH) is responsible for 5% of all strokes. Although usually asymptomatic, 5% of all strokes are caused by the rupture of an IA that results in SAH. IA can result in intraparenchymal or subdural hemorrhage, but it also accounts for 80%–85% of non-traumatic SAH (Van Gijn et al., 2007). Also, the Thyrotropin-releasing hormone tartrate (TRH-T) has been shown to have some efficacy in treating aneurysmal SAH-related extended consciousness disturbance (Meena et al., 2015). Current treatment strategies primarily involve invasive procedures like surgical clipping or endovascular coiling, which aim to prevent rupture by excluding the aneurysm from circulation (Zhao et al., 2018). While effective, these approaches carry inherent risks such as intraoperative complications, aneurysm recurrence, and long-term neurological deficits (Belavadi et al., 2021). Advances in molecular biology and pharmacology have spurred interest in small molecule inhibitors (SMIs) as a promising alternative therapy for IAs (Crane et al., 2024). These inhibitors target specific molecular pathways involved in IA pathogenesis, including inflammation, vascular remodeling, and oxidative stress (Ma et al., 2023). By modulating these pathways, SMIs aim to stabilize the aneurysm wall, promote endothelial repair, and reduce the risk of rupture without the invasiveness associated with traditional treatments (Crane et al., 2024). However, developing these inhibitors faces challenges such as optimizing their specificity and potency, determining optimal dosing regimens and delivery methods, and conducting robust clinical trials to establish their safety and efficacy compared to standard treatments. Despite these challenges, ongoing research holds considerable promise for advancing IA management through innovative pharmacological approaches. In this review, the pathogenesis mechanism in the formation and progression of IAs along with the role of SMIs in the treatment of IAs, their mechanisms of action, and the current status of preclinical and clinical research.

### 1.1 Evolution of IAs: a historical perspective

IA is a Cerebrovascular disease (CVD) that is well known for its high mortality and morbidity rate (Tang et al., 2020). IA is the dilatation of a brain-blood-supplying artery (Tromp et al., 2014) characterized by pathologic outpouchings of the cerebral vascular wall (Tutino et al., 2021). In the past 60 years, the development of endogenous animal models has helped to attempt to figure out the pathophysiology of IA and reveal the mechanism of IA formation. The first endogenous animal model was established in 1961 (White et al., 1961). In the following decades, techniques of IA animal model building steadily improved, and the rat model built by Hashimoto et al., in 1978 became a significant milestone, which succeeded in reproducing the common IA feature of humans in the rat model. When the early 20th century arrived, the application of IA animal models had become increasingly mature, and further development effectively helped researchers depict the mechanisms of AI in the development of targeted drugs through biological markers quantitative analysis (Tutino et al., 2021).

### 1.2 Incidence and prevalence of IAs

IA is unexpectedly common worldwide. According to international research, the incidence of IA globally may range between 0.65% and 7.0%, and it is estimated that 3% of the general population harbored these lesions (Rahmani et al., 2022). In the study of the prevalence of IA, it was found that genetic factors are believed to be an important reason for IA susceptibility. The family studies indicated a familial aggregation tendency toward IA. The genomic studies on Single, large families carried out several genome-wide DNA linkages results and found at least 6 genomic regions (1p34-36, 4q32, 7q11, 14q22) related to IA (Tromp et al., 2014). Several susceptibility loci for IA are identified and indicate a higher risk of susceptibility to IA in people with a family history of IA. However, the relationship between people without an IA family history but carrying susceptibility loci and developing IA has not been verified (Tromp et al., 2014). In addition, studies demonstrate genetic disorders, including Autosomal Dominant Polycystic Kidney Disease (ADPKD) and Ehlers-Danlos Syndrome (EDS) Type IV, are relevant to IA or SAH, which indicated approximately five times higher susceptibility than the general population (Cebral and Raschi, 2013). Other family genetic diseases, including Marfan’s syndrome, fibromuscular dysplasia, moyamoya disease, sickle cell disease, and arteriovenous malformations of the brain, were also demonstrated to be related to IA (Ajiboye et al., 2015).

Except for the congenital factors, Female gender, smoking, and hypertension are also identified as risk factors in the clinic. All these high-risk factors are believed to be related to inducing dysfunction of the vascular wall. Decreased estrogen levels in postmenopausal women induced compromise of vascular integrity (Jou et al., 2005). Moreover, smoking is a common trigger for inflammation in the arterial walls and, therefore, induces the weakens and predisposes the wall for aneurysm formation. Besides, the rupture induced by an increased aneurysm growth rate and inadequate wall repair caused by hypertension also leads to IA formation (Park et al., 2008).

### 1.3 Symptoms of IAs

Subarachnoid hemorrhage (SAH) is the major consequence of IA. Unlike the asymptomatic state when unruptured, the mortality rate of SAH due to ruptured IAs increases to as high as 50% as the disease progresses to its later stages. In the past few decades, the mortality rate of SAH did not show a significant increase. Different regions and populations are believed to be related to the incidence of aneurysmal SAH, and the number of patients that suffer from SAH in the US is estimated to affect about 30,000 individuals each year (Tromp et al., 2014). Besides, the aneurysm resulted in compression on the adjacent brain structure and convinced Middle cerebral artery aneurysms causing hemiparesis, visual field loss, or seizures; posterior communicating artery or basilar artery aneurysms causing third cranial nerve palsy; cavernous sinus aneurysms causing cavernous sinus syndrome; basilar distribution artery aneurysms causing brainstem compression; and, rarely, transient ischemic attack or cerebral infarction due to distal embolization of emboli in the aneurysm sac. Clinical also illustrated mild or severe headache, nausea, and vomiting (Broderick et al., 2009).

## 2 Pathophysiological factors contributing to IA formation

**FIGURE 1:**
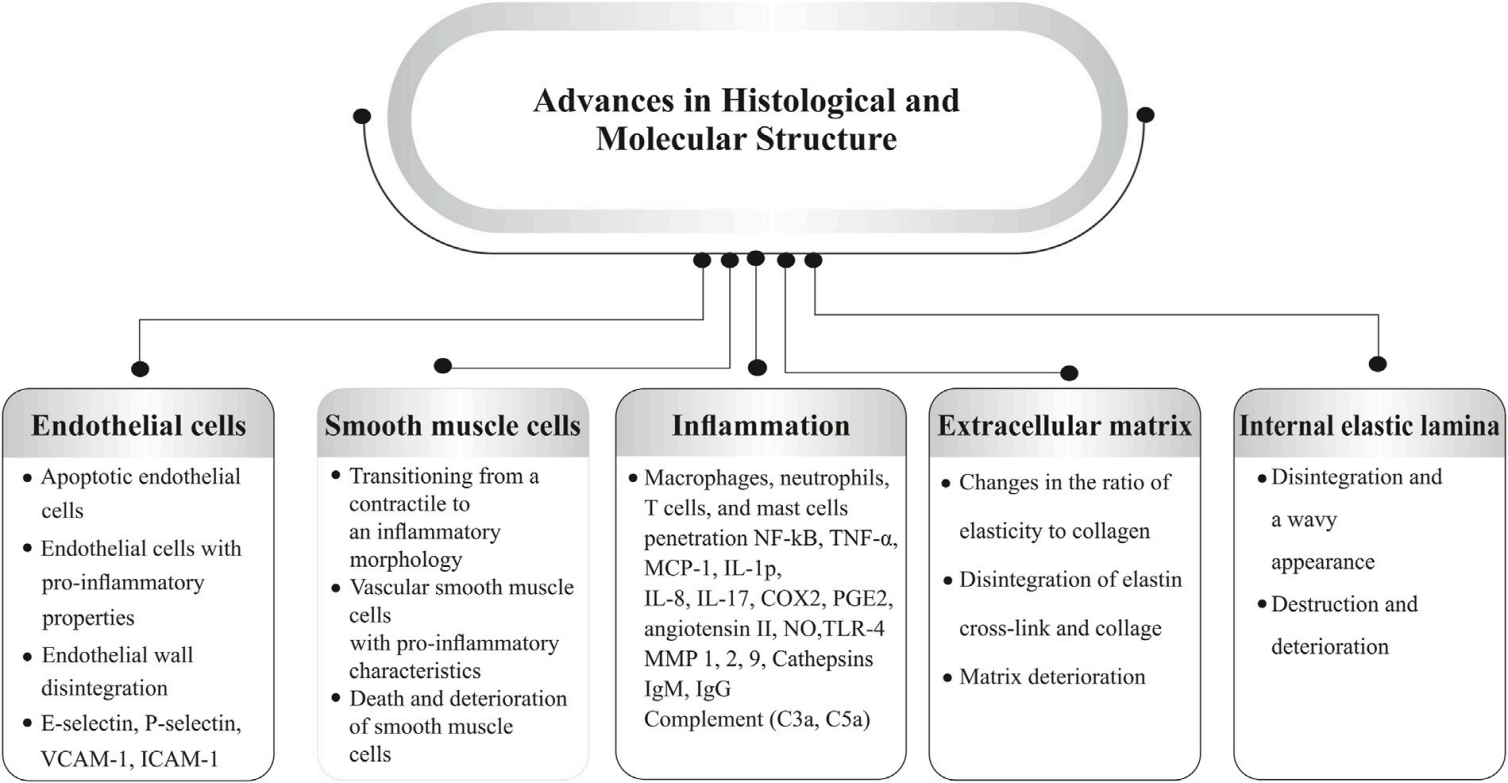
Pathophysiological factors involved in aneurysm formation (Abbreviations: VCAM-1, vascular cellular adhesion molecule-1; ICAM-1, intercellular adhesion molecule-1; TNFα, tumor necrosis factor-α; MCP-1, monocyte chemoattractant protein 1; IL, interleukin; COX, cyclooxygenase; PGE2, prostaglandin E2; NO, nitric oxide; TLR, toll-like receptor; MMP, matrix metalloproteinases).

According to the current research, the direct causative factors of IA formation are unclear but highly related to hemodynamics. Based on the tracking of the natural history of IA, mechanical properties of vascular walls are the key properties (Turjman et al., 2014). Blood flow plays an important role in aneurysm formation as a central process in many of the components. Bifurcations or sharp curves are preferred as the focal nature of lesions (Jersey and Foster, 2018) where inflammatory cells adhere, and how the endothelium and its cells respond to local inflammation (Penn et al., 2011). Cell proliferation and wall distension are considered combined acts to the growth of the IA. In contrast, blood pressure-induced tension exceeds aneurysmal wall strength, resulting from wall degeneration leading to IA rupture (Turjman et al., 2014). Apart from the hemodynamic effects, inflammation and calcification are also considered to contribute to IA development. On the one hand, inflammation is considered the key to the IA genesis, which results in flow-mediated endothelial dysfunction. Recent studies have evidenced that inflammatory cells (macrophages, T- & B-lymphocytes) can be commonly found in human IA (Jayaraman et al., 2008), while various inflammatory biological signals are verified as active experimental IA (Turjman et al., 2014). The pro-inflammatory cytokine, tumor necrosis factor-alpha (TNF-α), is highly expressed in the IA and initiates damage to the endothelium, smooth muscle cells (SMC), and internal elastic lamina (IEL) (Jayaraman et al., 2008). On the other hand, as early as the early 1900s, the phenomenon of calcification was described in IA by neurosurgeons (Rahmani et al., 2022). Calcification occurs primarily in arterial, smooth muscle cells and finally induces IA calcification. The histopathological studies indicated that remodeling is undergone during the period of IA formation. Initially, the normal arterial wall undergoes hypertrophy and turns into a thinning process, with ruptured aneurysms often exhibiting a hyalinized remnant of the wall in certain areas (Frosen et al., 2004). Atherosclerotic calcification plays an important role in osteogenic differentiation, whereas smooth muscle cells are generated into a phenotypic switching that contributes to aneurysm formation (Rahmani et al., 2022). Other than the factors mentioned above, infective and traumatic aneurysms are rare lesions in IA but are still considerable in penetrating head injuries (Kumar and Kitchen, 1998). IA is associated with significant structural changes in the vascular wall. Understanding the pathophysiological factors involved can help identify patients who may be at risk. While the precise mechanisms behind the formation, growth, and rupture of cerebral aneurysms are still unclear, several key factors have been proposed, including defects and degeneration of the extracellular matrix, hemodynamic stress, and inflammatory responses, all of which contribute to the weakening of the arterial wall. These factors are closely linked to one another (Rodríguez et al., 2008; Chalouhi et al., 2012). SMIs may be able to treat a number of pathophysiological issues related to the onset and progression of intracranial aneurysms. These inhibitors work to stabilize aneurysms, reduce inflammation, and stop rupture by focusing on these particular pathways (Gruszka et al., 2018). A summary of the structural component’s alterations is shown in Figure 1.

### 2.1 Deterioration and defects in the extracellular matrix

The extracellular matrix, a dynamic structure, is constantly remodeled by vascular cells, with smooth muscle cells playing a crucial role in its mechanical strength. Elastin and collagen cross-linking are essential for artery strength. Damage to the extracellular matrix can lead to IAs if an imbalance between MMPs (Matrix metalloproteinases) and inhibitors occurs (Rodríguez et al., 2008). Deficiency in dietary copper during development can reduce LOX activity, compromising blood vessel wall integrity and potentially leading to aneurysm formation in adulthood, as demonstrated in a study in mice during development (Gacheru et al., 1990; Jung et al., 2016). A study found that surviving mice’s thoracic aortae dilate with disorganized elastic fibers, leading to fusiform and saccular aneurysms. Early dietary habits may influence aneurysm prevalence, particularly in cow’s milk or low-copper infant formulas. Connective tissue disorders like osteogenesis imperfecta, Ehlers-Danlos syndrome, and Marfan syndrome are often linked to IAs (Lönnerdal et al., 1985; Lönnerdal, 1998; Cury et al., 2013).

### 2.2 Hemodynamic stress

IAs are common in arterial junctions like the anterior communicating artery, posterior communicating artery, middle cerebral artery bifurcation, and basilar artery bifurcation, where arterial wall shear stress is highest, leading to endothelial cell damage, smooth muscle cell degeneration, media thinning, and aneurysm development (Metaxa et al., 2010; Baharoglu et al., 2014; Zhang et al., 2018). Shear stress is a key factor in aneurysm development in predisposed individuals. Flow diverters reduce stress, but computational fluid dynamics reveal complex hemodynamics. High wall stress is linked to aneurysm formation, while low stress predicts rupture. Smoking increases aneurysm prevalence and rupture risk (Seshadhri et al., 2011; Zhou et al., 2017).

### 2.3 Inflammatory response

IAs are caused by inflammation and degeneration of the medial wall, triggered by various immunological factors. Elevated hemodynamic stress affects endothelial function, leading to inflammatory cell infiltration and activation in the tunica media. Factors like monocyte chemoattractant protein 1, NF-κB, angiotensin II, prostaglandin E2, and prostaglandin E receptor subtype 2 enhance this inflammatory response, causing phenotypic changes in smooth muscle cells, pro-inflammatory responses, matrix remodeling, and apoptosis (Aoki and Nishimura, 2010; Chalouhi et al., 2013; Fennell et al., 2016). A study found that elevated cytokines and chemokines in IA plasma weaken vessel walls, leading to aneurysmal dilation and rupture (Cao et al., 2010).

## 3 Contemporary approaches to diagnosis, prevention, and treatment of IAs

### 3.1 Diagnosis

Radiographic evaluation is critical for the initial diagnosis of cerebral pathology. Plain film X-rays are used to be an efficient way to provide IA images to assist in diagnosis (Voigt et al., 1975; Rahmani et al., 2022). In the 1970s, computed tomography (CT) was introduced into IA Imaging and became a standard for diagnosis (Rahmani et al., 2022). With the development of radiographic technology, CT angiography (CTA), magnetic resonance angiography (MRA), and angiography by direct intra-arterial catheterization (catheter angiography) are more common in clinical diagnosis currently. The preceding two techniques are more applicable for screening of unruptured aneurysms because of their noninvasive nature and high rate of accuracy and specificity. Catheter angiography, however, is considered the gold standard for IA diagnosis, which can provide a detailed evaluation of the aneurysm with other vessels. An effective method for evaluating unruptured intracranial aneurysms (UIAs) is high-resolution vascular wall imaging or HR-VWI. The contrast enhancement of the aneurysmal wall cannot be quantified using any established technique. Signal intensity is an objective way to quantify contrast enhancement, or it may be judged subjectively. Evaluated several UIA enhancement quantification techniques and discovered that CRstalk was the most trustworthy objective technique to distinguish aneurysms larger than 7 mm (Roa et al., 2020). Ultrasmall superparamagnetic iron oxide particles (USPIOs) are being investigated as a noninvasive cerebral aneurysm screening tool in the setting of inflammation. USPIOs are hydrophilic-coated iron oxide cores that accumulate in macrophages and can be employed as MRI contrast agents for tissues that are actively inflamed. A USPIO authorized for treating anemia in patients with chronic renal disease, ferumoxytol has the potential in neuroimaging to identify unstable brain aneurysms. Research reveals that it may be used to detect macrophages in aneurysm walls using enhanced magnetic resonance imaging (MRI). Early uptake is linked to aneurysm instability and elevated levels of inflammatory molecules (Iqbal et al., 2024). Unfortunately, the higher price and invasiveness are still the primary reasons hindering its current use as the preferred screening method in clinical practice (Ajiboye et al., 2015).

### 3.2 Preventive measures

Compared to the SAH, which is the major fatal consequence, the unruptured IA reared induced a significant impact on human health. For this reason, the most important preventive measure is to prompt the obliteration of IA, especially ruptured aneurysm, and accordingly reduce the probability of rebleeding. Based on the 2023 guideline for the management of patients with aneurysmal subarachnoid hemorrhage there are three major strategies in clinical healthcare: reduction of blood pressure (beware of sudden vibration in blood pressure to result in a poorer prognosis), immediate anticoagulation reversal, anti-fibrinolytic therapy (Hoh et al., 2023).

### 3.3 Therapy

Currently, the basic strategy of IA treatment includes surgical, endovascular therapy, and combination therapy, as mentioned below in Table 1. The former typically refers to a microsurgical clipping with or without bypass techniques, and the second strategy includes coiling, balloon- or stent-assisted coiling, or intravascular flow diversion and intravascular flow disruption. With the development of IA treatments, surgical methods become specialized in anterior circulation treatment, whereas endovascular techniques, including Balloon-assisted coiling (BAC), stent-assisted coiling (SAC), and flow diversion, are more popular in treating posterior circulation or IAs with complex morphologies such as large, wide-necked, and fusiform IAs (Lee et al., 2022). However, single-treatment regimens alone cannot meet all clinical needs. Hence, the combination of these two methods is also very common today.

**TABLE 1:** Common therapies applied for IA treatment.

Treatment	Techniques	Principle and indications
Endovascular	Simple coiling	First employed in 1990, this technology enhances exposure to blood through its hydrophilic gel coat and minimizes dead space.
	Balloon-assisted coiling (BAC)	Utilized in wide-neck IA, this technique involves inflating a flexible balloon within the parent vessel's lumen. This temporary narrowing of the aneurysm's neck facilitates precise coil placement inside the aneurysm, thereby preventing its protrusion into the parent vessel.
	Stent-assisted coiling	Initially employed in the treatment of IA in 2002, closed-cell stents are distinguished by small spaces between their struts, whereas open-cell stents feature larger uncovered gaps.
	Double microcatheter technique	Utilizes for IA with complex structure and not suitable for simple spring coils.
	Flow diverters	Flexible tubular structures with less porous mesh promote thrombus formation in intracranial aneurysms by causing blood stasis, facilitating aneurysm regression.
	Intrasaccular flow disruptors	Woven Endoluminal Bridge (WEB) is a treatment for bifurcation intracranial aneurysms, redirecting flow away from the aneurysmal sac, promoting stagnation, and facilitating thrombosis.
Surgical techniques	Microsurgical clipping	IA surgery utilizes an open craniotomy to separate the aneurysm from surrounding tissue, improve neck visibility, and permanently close it off with a metallic clip.
	Bypass techniques	The ligation of the IA's parent artery, also known as extracranial-to-intracranial (EC-IC) or intracranial-to-intracranial (IC-IC), leads to obliteration.
Hybrid techniques	Microsurgical treatment post-endovascular therapy	Microsurgical treatment is limited in ruptured IA, but microsurgical clipping can resolve unfavorable anatomy after initial coil embolization.
	Endovascular therapy post-microsurgical treatment	Stable coil constructs for wide-based morphology IA are challenging to create, but they can embolize the narrow neck due to limited surgical corridor after IA neck clipping.

In 1933, Norman Dott’s first attempt to wrap a ruptured IA is illustrated as the first Surgery of IA based on the written records (Dott, 1933). In 1938, Walter Dandy performed the first case of obliterative clipping of an IA (Dandy, 1938). In 1975, Yasargil and Fox detailed the classic microscopic-assisted open techniques for clipping IAs, notably the personal craniotomy (PTC). This approach enabled secure and efficient access to the circle of Willis through the Sylvian fissure, with minimal need for frontal and temporal lobe retraction (Mg and Fox, 1975). With the International Subarachnoid Aneurysm Trial (ISAT) result published in 2002, endovascular techniques, which replaced Surgery, became more popular and highly accepted because of their safety and efficiency (Molyneux et al., 2005; Lee et al., 2022).

In addition to the therapies mentioned above, drug treatment is a crucial component of IA management. Calcium antagonist is a common solution for hypertension that can also be used to reduce the negative impact caused by SAH-induced Ischemia. Oral nimodipine 60 mg every 4 h is applied to clinical practice (Mees et al., 2007). Research indicated that Aspirin administration significantly reduces the risk of IA rupture. Still, whether it is safe to use in the clinical is not well-studied since potential risks (Ajiboye et al., 2015). After analyzing data from patients who had many minor aneurysms (≤5 mm), Zanaty et al. discovered that aspirin was significantly associated with a lower aneurysm growth rate in 146 individuals who had 229 aneurysms and were followed up for at least 5 years. Adherence to the aspirin regimen was recorded in the medical record, and aspirin usage was defined in this study as ≥81 mg daily (Zanaty et al., 2019).

## 4 Requirement for new IA drug therapy

Considering that non-drug therapies currently remain the mainstream approach for treating IA, the complications, including rupture resulting in massive cerebral hemorrhage and ischemic stroke, are undeniable for these therapies (Radić et al., 2021). Moreover, whether small, symptomless, unruptured aneurysms are suitable for applying neurosurgery intervention or endovascular embolization is still questionable (Gruszka et al., 2018). The existing drug therapy only provides symptomatic relief, lacks a curative solution, and still lacks adequate data to support building an interaction for clinical application. Consequently, a new inspiring drug therapy is urgently needed.

SMIs function by attaching to the “pocket” on the surface of target proteins to prevent them from functioning. Since they are smaller than antibodies, SMIs can bind a larger variety of extracellular and intracellular targets. Additionally, although antibodies are supplied intravenously or subcutaneously, the majority of SMIs are taken orally. Furthermore, several SMIs can cross the blood-brain barrier and regulate intracranial lesions. The first described kinase medicines appeared in the 1990s, although chemicals that inhibit kinases have been utilized as medications since the 1930s. First among them was fasudil, a small molecule Rho kinases (ROCKs) inhibitor that was licensed in Japan in 1995 to treat cerebral vasospasm (Lightfoot et al., 2018; Liu et al., 2022). Numerous medical advances and unmet medical needs have been made possible by small molecules, saving numerous lives in the process. Furthermore, small molecules have proven essential for understanding the cellular mechanisms of disease as therapeutic agents in biomedical research.

Over the past century, conventional small-molecule medicines have driven pharmacological research. SMIs are among the main targeted treatments available for cancer and several other life-threatening pathologies. Several efforts have been made to produce additional SMIs because of their benefits, which include the capacity to reach the central nervous system, a wide variety of targets, and easy dosing. The use of SMIs in adjuvant, first-, and post-line therapy has been approved (Liu et al., 2022; Beck et al., 2022). SMIs are important in several areas of medicine, including prophylaxis, therapy, and diagnostics.

### 4.1 Classes of SMIs

SMIs are a group of chemical substances that block certain enzymes, receptors, or other proteins involved in disease mechanisms to interfere with particular biological processes. The molecular weight of small molecule inhibitors is usually less than 500 Daltons. They may readily permeate cells and tissues due to their tiny size. Compared to bigger biological molecules like proteins or antibodies, they are often made up of simpler chemical structures and frequently have less complicated components (Southey and Brunavs, 2023). These inhibitors are classified into several therapeutic groups based on their specific targets and mechanisms of action. For example, enzyme inhibitors include Kinase Inhibitors, which target kinases involved in cell signaling; Protease Inhibitors, which block proteases necessary for viral development or protein processing; and Phosphodiesterase Inhibitors, which reduce cyclic nucleotide levels by inhibiting phosphodiesterases (Roskoski Jr, 2016). Receptor inhibitors consist of Angiotensin Receptor Blockers, used for managing hypertension and heart failure, and Histamine Receptor Antagonists, which block histamine receptors to address allergies and acid reflux (Barreras and Gurk-Turner, 2003). Ion channel blockers alter cellular electrical activity, including Calcium Channel Blockers, used for hypertension and angina, and Sodium Channel Blockers, used for arrhythmias (Katz et al., 1984). Nuclear Receptor Modulators feature Steroid Receptor Modulators, which are employed in breast cancer treatment, and Thyroid Receptor Modulators, used for thyroid hormone replacement (Lonard and O’malley, 2012). Additionally, antimetabolites are predominantly used in cancer therapy, while Histone Deacetylase Inhibitors impact gene expression by modifying histone acetylation (Giannini et al., 2012). These groups illustrate the wide range of mechanisms through which small molecule inhibitors deliver their therapeutic effects by targeting various cellular functions and disease processes.

### 4.2 Role of SMIs in diagnosis

SMIs play a key role in finding biomarkers linked to certain diseases and can aid in identifying diagnostic biomarkers by focusing on particular pathways or enzymes that are dysregulated under particular circumstances (Yi et al., 2018). Certain small molecules can be engineered to function as agents of imaging. To help with diagnostic imaging, SMIs tagged with radioactive tracers, for instance, can be utilized in positron emission tomography (PET) scans for identifying certain molecular targets in tissues. SMIs are utilized in infectious diseases and cancer to find resistance mechanisms or mutations. Healthcare professionals can anticipate or keep track of the emergence of resistance in infections or cancer cells by analyzing the interactions that certain inhibitors have with target molecules (Crisan et al., 2022).

## 5 Preventive strategies for SMIs

SMIs can be used proactively to prevent the advancement of certain diseases or lower the probability of developing diseases. For instance, in cancer, high-risk patients can be treated with inhibitors that target oncogenic pathways to stop the growth or beginning of tumors (Chehelgerdi et al., 2023).

### 5.1 Antiviral prophylaxis

SMIs can function as antiviral drugs in infectious disorders that inhibit the propagation of viruses. This strategy is especially useful during epidemics or outbreaks when prompt implementation of preventative measures is crucial (Chuang and Buchwald, 2022).

### 5.2 Therapeutic functions

SMIs have a major role in cancer treatment. They target altered proteins or certain signaling pathways that promote the development of tumors. Protease inhibitors, such as bortezomib for multiple myeloma, and tyrosine kinase inhibitors, such as imatinib for chronic myeloid leukemia, are two examples. Treatment for infectious disorders, especially anti-viral and antibacterial medications, has been transformed by SMIs. They target vital proteins or enzymes found in infections, including penicillin and HIV protease inhibitors. SMIs may target enzymes implicated in neurodegeneration or amyloid plaque production in disorders such as Parkinson’s and Alzheimer’s to reduce symptoms or prevent the disease’s development. Targeting enzymes involved in glucose metabolism or lipid synthesis, SMIs can modify metabolic pathways and provide possible therapies for conditions such as diabetes and obesity (Al-Hussaini and DiPersio, 2014).

For Personalized medicine, new developments in proteomics and genomes will make it possible to create SMIs that are specific to each patient’s profile, maximizing the benefits of treatment while reducing side effects. For Combination medicines, more SMI-based combination medicines that target many pathways at once or combine inhibitors with other forms of treatment, like immunotherapy, are probably in store for the future. Innovations in Drug Delivery targeted drug delivery methods, and nanotechnology may improve the specificity and efficacy of SMIs, maximizing their therapeutic potential and reducing off-target effects. For Emerging Targets as disease processes are further investigated, novel molecular targets for SMIs will be found, hence broadening the therapeutic range of disorders for which they may be used (Serra et al., 2023). SMIs offer a targeted approach to modifying the molecular pathways involved in IA pathogenesis.

Some of the key targets and their mechanisms of action include:

#### 5.2.1 Matrix metalloproteinase (MMPs) inhibitor

MMP inhibitors have been researched as a potential therapy for neurological diseases, cancer, and cardiovascular disease since the MMP family of proteins has biological roles essential to human health and medical conditions. Initial clinical trial results with MMP inhibitors from the first generation were unsatisfactory. Nevertheless, novel alternatives and paths for the development of MMP-focused treatments are now available owing to the expanding awareness of the complexity of MMP function in disease as well as increasing knowledge of the design of the MMP protein and its regulation of activity (Abdul-Hussien et al., 2009; Kumar et al., 2019).

Testing MMPIs for the treatment of invasive or metastatic tumors has not always produced the anticipated favorable outcomes, but it has aided in the advancement of MMPI development. On the other hand, compared to inflammation, tumors appear to have higher genetic variability and instability, suggesting that treatment outcomes may be less predictable. MMP inhibitors prevent the degradation of ECM components, thereby maintaining vessel wall integrity. For instance, Doxycycline, an FDA-approved antibiotic, has shown promise in inhibiting MMP activity and reducing aneurysm progression in animal models. Matrix metalloproteinases contribute to extracellular matrix degradation in vessel walls, weakening and predisposing to aneurysm formation. Inhibitors such as Doxycycline have demonstrated efficacy in stabilizing aneurysm walls by inhibiting MMP activity (Hu et al., 2007).

#### 5.2.2 Anti-inflammatory agents

These agents target pro-inflammatory pathways to reduce inflammation and subsequent vessel wall degradation. Statins, such as simvastatin, exhibit anti-inflammatory effects by inhibiting the NF-κB pathway, reducing the expression of pro-inflammatory cytokines. Chronic inflammation plays a pivotal role in cerebral aneurysm pathogenesis. SMIs targeting pro-inflammatory cytokines (e.g., TNF-alpha, IL-6) and signaling pathways like NF-κB show promise in reducing inflammation and preventing aneurysm growth (CF, 1992; Ueda et al., 1994; Collins et al., 1995; Khan et al., 2023).

#### 5.2.3 Modulating vascular smooth muscle cells (VSMCs)

Remodeling of the vascular matrix occurs concurrently with aneurysm development. Vascular remodeling is widely recognized to be a process that depends heavily on the phenotype of VSMCs for the creation and breakdown of extracellular matrix (ECM). Vascular endothelial cells (VECs), VSMCs, and inflammatory cells were among the many cellular and molecular elements that intricately interacted throughout the development of IA. Vascular remodeling, which aided in the genesis, growth, and rupture of aneurysms, was mediated in large part by VSMCs. VSMCs altered from having a contractile phenotype to a range of phenotypes, including osteogenic, foamy, and macrophagic morphologies, in response to different triggers. Throughout one or more phases of IA, these various phenotypes served diverse purposes. As a result, VSMC phenotypic traits can serve as useful markers for IA early detection, clinical staging, and rupture evaluation. Inhibition of Rho-Kinase (ROCK) reduces VSMC apoptosis and inflammation, thereby stabilizing the aneurysm wall. Fasudil, a ROCK inhibitor, has shown efficacy in reducing aneurysm formation and progression in preclinical studies (Tang et al., 2019; Wang et al., 2023).

#### 5.2.4 Angiotensin II receptor blockers (ARBs)

ARBs reduce blood pressure and have anti-inflammatory effects, potentially preventing aneurysm formation. Vascular remodeling and inflammation are significant aspects of the pathogenesis of IAs, and angiotensin can regulate these mechanisms. A retrospective analysis of patients in China discovered that the use of a blood pressure drug called a renin-angiotensin-aldosterone system (RAAS) inhibitor potentially significantly decreases the risk of IA rupture in individuals with hypertension and an IA. Losartan has been investigated for its protective effects against aneurysm development in experimental models (Barreras and Gurk-Turner, 2003; Schmieder, 2005).

#### 5.2.5 Modulation of endothelial function

It is well established that endothelial cell (EC) dysfunction plays a role in the development of IA. Evidence suggests that the initial event in the genesis of an IA is damage or injury to the EC layer. Endothelial dysfunction is a hallmark of aneurysm development. Small molecules that enhance endothelial nitric oxide synthase (eNOS) activity or inhibit endothelial cell apoptosis, Hemodynamic stress, oxidative stress, estrogen imbalance, and endothelial cell-to-cell junction impairment are among the connected factors underlying EC dysfunction can improve vascular integrity and reduce aneurysm risk (Sheinberg et al., 2019).

## 6 Preclinical and clinical investigations

Different preclinical studies have shown the efficacy of synthetic matrix metalloproteinase inhibitors (SMIs) in reducing aneurysm growth and preventing rupture. Doxycycline and statins, for example, have been effective in inhibiting MMP activity and reducing aneurysm growth rates in rodent models. Li et al. found that aspirin reduced CA size, macrophage infiltration, and the expression of MMP-2 and MMP-9 in male Sprague-Dawley rats while reversing the upregulation of NF-κB, MCP-1, and VCAM-1 (Li et al., 2015). Similarly, Aoki et al. reported that simvastatin increased media thickness and decreased CA size and inflammatory markers (Aoki et al., 2008), while Tada et al. noted that low-dose pravastatin reduced endothelial damage and aneurysm formation, but higher doses of pravastatin and simvastatin promoted aneurysmal growth and rupture (Tada et al., 2011). Jin et al. observed overexpression of MMP-2 and MMP-9 in ruptured aneurysms, with higher MMP to TIMP ratios (Jin et al., 2007). Nuki et al. found that Doxycycline reduced aneurysm incidence, particularly in MMP-9 knockout mice (Nuki et al., 2009), while Makino et al. showed that minocycline and Doxycycline reduced rupture rates in hypertensive mice with elastase-induced aneurysms (Makino et al., 2012). Additionally, Yagi et al. demonstrated that ibudilast suppressed aneurysm formation by down-regulating PDE-4 and reducing macrophage migration (Yagi et al., 2010), and Chen et al. confirmed that simvastatin decreased aneurysm size and inflammatory cytokine levels in rats (Chen et al., 2022). These findings suggest that targeting inflammation and matrix remodeling can effectively modulate CA progression. Bigatel et al. discovered that the growth of aneurysms in this rat model was restricted by MMP inhibition with BB-94 (batimastat), a particular inhibitor of MMPs. In addition to acting as a direct pharmacologic inhibitor of MMPs, BB-94 also seems to function by interfering with the inflammatory response that aneurysms cause (Bigatel et al., 1999). Several forms of statins, calcium channel blockers, and angiotensin II receptor blockers are potential medications for the preventative treatment of unruptured IAs, according to clinical research that included 310 patients with ruptured and 887 individuals with unruptured IAs (Shimizu et al., 2021).

Furthermore, clinical studies have also explored the impact of medications on IAs. Atorvastatin has shown promise in reducing aneurysm wall enhancement and inflammation markers in patients with unruptured IAs. A phase 2, single-center, randomized, controlled, double-blind clinical study is called the Statin Treatment for Unruptured Intracranial Aneurysms Study. Sixty individuals with aneurysm wall augmentation and unruptured IAs (Li et al., 2020; Kang et al., 2022). Aspirin usage has been linked to a lower risk of aneurysm rupture (Hasan et al., 2011; Hostettler et al., 2018; Can et al., 2018a) and reduced growth rates of small IAs (Zanaty et al., 2019), although it may increase re-rupture risk post-rupture (Can et al., 2018b). Combining aspirin with statins has been associated with unruptured IAs (Terceño et al., 2021), while statins, calcium channel blockers, and angiotensin II receptor blockers have been linked to reduced rupture risk (Shimizu et al., 2021). Aspirin use has also been associated with decreased aneurysm enhancement in high-resolution vessel wall imaging (Roa et al., 2020). Dexamethasone, despite being linked to higher rates of hyperglycemia and infections, has shown favorable outcomes in microsurgically treated patients (Schürkämper et al., 2004; Czorlich et al., 2017), and fludrocortisone has been effective in reducing sodium balance issues in SAH patients (Hasan et al., 1989). Elevated pro-inflammatory cytokines in cerebrospinal fluid have been noted in patients with unruptured IAs (Kamińska et al., 2022), and biomarkers such as plasma miR-143/145 and serum MMP-9 have been associated with ruptured IAs (Feng et al., 2018). Inflammation has a critical role in the development, progression, and rupture of IAs, according to findings from clinical studies and animal models. IAs can be mediated by inflammation by hemodynamic stress (Zanaty et al., 2019). Usually brought on by a hemodynamic shock, inflammation of the aneurysmal wall can lead to aneurysm remodeling and rupture, localized cytokine activation, endothelial and vascular smooth muscle cell dysfunction, and extracellular matrix degradation (Roa et al., 2020). It was also demonstrated that inflammation was, at least in part, responsible for the hypertension-induced IAs in rats. Ultimately, it was determined that inflammation was responsible for the development, expansion, and rupture of aneurysms in rat models because of the extracellular matrix-degrading proteolytic enzymes (matrix metalloproteinase–2 and –9) released by macrophages (Zanaty et al., 2019). Treatment for small- and medium-sized cerebral aneurysms can be achieved safely and effectively with the Medtronic pipeline embolization device (PED). The Food and Drug Administration gave PED approval in 2011. Initially, it was only used to treat massive intracranial aneurysms with broad necks, ranging from petrous to superior hypophyseal portions of the internal carotid artery (ICA) (Hanel et al., 2020). These studies underscore the potential of various pharmacological interventions in managing cerebral aneurysms by targeting inflammation, matrix remodeling, and other underlying mechanisms.

## 7 Drug delivery systems for SMIs

Intracranial aneurysm treatment involves various pharmacological approaches, each focusing on different aspects of the illness and its consequences. Calcium channel blockers like Nimodipine prevent aneurysm rupture and manage vasospasm by decreasing calcium influx in smooth muscle and neurons. Mannitol lowers intracranial pressure and controls subsequent vasospasm, especially after subarachnoid hemorrhage. Labetalol treats hypertension by inhibiting beta- and alpha-adrenergic receptors. Statins like atorvastatin decrease cholesterol and stabilize aneurysm walls, slowing aneurysm progression as depicted in Table 2. These medications are essential in an all-encompassing treatment plan tailored to individual needs.

**TABLE 2:** Drugs and their mechanistic targets for treating various conditions of IA.

S. No.	Condition	Drug	Drug class	Target
1	Preventing Aneurysm Rupture	Nimodipine	Calcium Channel Blocker	Calcium channels in smooth muscle and neurons
2	Reducing Intracranial Pressure	Mannitol	Osmotic Diuretic	Blood-brain barrier and cerebral edema
3	Preventing Vasospasm	Calcium Channel Blockers (e.g., Nimodipine)	Calcium Channel Blocker	Calcium channels in smooth muscle
4	Managing Hypertension	Labetalol	Beta-Blocker/Alpha-Blocker	Alpha and beta-adrenergic receptors
5	Reducing Risk of Aneurysm Growth	Statins (e.g., Atorvastatin)	Statin	HMG-CoA reductase
6	Anticoagulation (after aneurysm repair)	Heparin	Anticoagulant	Antithrombin III
7	Long-Term Anticoagulation	Warfarin	Anticoagulant	Vitamin K epoxide reductase

Recent research on treatments for aneurysmal subarachnoid hemorrhage (aSAH) and intracranial aneurysms shows that combining aggressive angioplasty with continuous nimodipine reduction and nimodipine infusion improves recovery, with 95.8% of patients achieving good outcomes (Chen et al., 2018). Another study of 124 patients found that higher doses of mannitol (1.0 and 1.5 g/kg) were more effective in enhancing brain relaxation during surgery, resulting in satisfactory outcomes for 67.7% and 64.5% of patients (Seo et al., 2016). A study also found that 12 months of daily atorvastatin significantly reduced aneurysm wall enhancement, improved aneurysmal morphology, and decreased inflammation markers in 60 unruptured IA patients (Li et al., 2020). Conversely, a study on 109 patients with ruptured cerebral aneurysms found that higher preoperative modified Fisher grades and external ventricular drainage were key predictors of rebleeding rates (Choi et al., 2021). Additionally, a study involving 4,696 patients with 6,403 aneurysms found elevated INR strongly associated with aneurysm rupture in non-anticoagulated patients, while warfarin-induced higher INR reduced rupture risk (Can et al., 2018a). Lastly, a patient with Fisher grade 3 subarachnoid hemorrhage experienced severe hypotension and bradycardia after intravenous labetalol infusion. Despite initial effectiveness, it led to a significant drop in blood pressure, requiring intensive vasopressor therapy (Jivraj et al., 2006). These studies provide valuable insights into the management of aneurysms and aSAH, emphasizing the impact of different treatments and risk factors on patient outcomes.

The therapy landscape for IAs has completely changed due to recent developments in drug delivery technologies, especially when it comes to SMIs (Ezike et al., 2023). These inhibitors, which target key molecular pathways involved in aneurysm formation and progression, require precise delivery mechanisms to ensure efficacy and minimize systemic adverse effects (Wang et al., 2015). Nanoparticle-based carriers, liposomes, and polymeric micelles have emerged as promising platforms, providing targeted delivery, sustained release, and enhanced permeability across the blood-brain barrier (BBB). These systems improve the bioavailability and therapeutic index of SMIs, allowing for localized treatment of aneurysms with reduced toxicity (Yetisgin et al., 2020). Certain small molecule inhibitors can cross the blood-brain barrier and regulate intracranial lesions. In a phase I clinical study, paxalisib, a brain-penetrant dual PI3K/mTOR inhibitor, was shown to be able to pass the blood-brain barrier (NCT01547546) (van den Broek et al., 2022). Paxalisib is now undergoing further studies for cancers that have primary and secondary brain metastases, such as glioblastoma and breast cancer that has spread to the brain. Moreover, surface modifications and ligand conjugation techniques further enhance the targeting capabilities, enabling specific binding to aneurysm sites and optimizing therapeutic outcomes. As research in this area progresses, drug delivery systems for SMIs hold great potential in offering safer and more effective interventions for patients with IAs (Li et al., 2023). Developing poses significant challenges primarily due to the complex pathophysiology and unique anatomical location within the brain. One of the major hurdles is achieving specific targeting of molecular pathways involved in aneurysm formation and progression without causing off-target effects or disrupting normal brain function (Toader et al., 2023). BBB presents another formidable obstacle, limiting the delivery of therapeutic agents to the aneurysm site while maintaining sufficient therapeutic concentrations (Partridge et al., 2022). Additionally, the heterogeneous nature of aneurysms necessitates a personalized medicine approach, as individual variations in genetic predispositions and environmental factors can influence treatment efficacy (Toader et al., 2023). Furthermore, the lack of a comprehensive understanding of the molecular mechanisms underlying aneurysm development complicates the identification of suitable targets for inhibition (Tromp et al., 2014). Overcoming these challenges requires innovative strategies in drug design, delivery systems, and biomarker identification to advance effective small-molecule therapies for IAs.

In current drug delivery technologies, nanoparticles and liposomes show promising potential for the treatment of IAs (Naqvi et al., 2020). Due to their small size and surface modifications, nanoparticles can potentially penetrate the BBB and target specific sites within the brain, including aneurysms, while minimizing systemic adverse effects (Pinheiro et al., 2021). Encapsulation of drugs within liposomes enhances their stability and bioavailability, facilitating controlled release and prolonged circulation in the bloodstream, which is critical for sustained therapeutic effect in treating aneurysms (Fukuta et al., 2022). Moreover, these technologies enable precise delivery of therapeutic agents such as anti-inflammatory drugs, antioxidants, or gene therapy vectors directly to the aneurysm site, addressing the underlying pathophysiological mechanisms without affecting healthy brain tissue (Liu et al., 2024).

## 8 Approaches for enhancing BBB penetration

Effective treatment of IAs necessitates the development of strategies to penetrate the BBB, a selective permeability barrier that protects the brain but also limits drug delivery (Dong, 2018). One approach involves the use of focused ultrasound (FUS) in conjunction with microbubbles, which temporarily disrupts the BBB, allowing therapeutic agents to reach the aneurysm site (Fisher and Price, 2019). Another strategy employs receptor-mediated transcytosis, leveraging endogenous transport mechanisms by conjugating drugs to ligands or antibodies that bind to receptors on BBB endothelial cells, facilitating their transport across the barrier (Jones and Shusta, 2007). Nanoparticle-based delivery systems also offer potential, with surface modifications enabling nanoparticles to exploit BBB transport pathways or transiently open tight junctions (Lim et al., 2024). Liposomal carriers can be engineered to enhance BBB penetration through similar mechanisms. Advances in these strategies aim to achieve targeted, efficient, and safe delivery of therapeutics to the brain, addressing the challenge of treating IAs while preserving the protective function of the BBB (Juhairiyah and de Lange, 2021).

## 9 Conclusion

IAs are difficult to understand and treat because of their complicated pathophysiology, which includes genetic, environmental, and hemodynamic variables. Current preclinical models frequently fail to precisely reproduce human IAs, restricting the translation of experimental discoveries into therapeutic applications. BBB is a significant impediment to successful medication delivery, further complicating therapeutic efforts. SMIs that target particular biochemical pathways, like as inflammation and apoptosis, show potential in the treatment of IA, but they come with hazards such as systemic toxicity and neurotoxicity. Targeting the underlying pathophysiological mechanisms, these SMI technologies allow for the precise administration of therapeutic medicines, like as antioxidants, gene therapy vectors, or anti-inflammatory medications, directly to the aneurysm site. Recent advances in drug delivery technologies, customized medical techniques based on genetic profiling, and the use of computer modeling are propelling innovation in IA research. Collaboration is critical for developing these techniques and overcoming problems in clinical trial design, assuring comprehensive examination of SMIs for their safety and efficacy in managing IAs effectively. Subsequent investigations ought to concentrate on enhancing preclinical models to more accurately represent human circumstances, developing medication delivery methods to surmount the blood-brain barrier, and utilizing genetically profiled tailored medicine strategies. It is also important to explore innovations in computer modeling, and more cooperation is required to tackle problems in clinical trial design and fully assess the safety and effectiveness of SMIs.
